# What Is the Role of Imaging at Primary Diagnostic Work-Up in Uterine Cervical Cancer?

**DOI:** 10.1007/s11912-019-0824-0

**Published:** 2019-07-29

**Authors:** Ingfrid S. Haldorsen, Njål Lura, Jan Blaakær, Daniela Fischerova, Henrica M. J. Werner

**Affiliations:** 1 Mohn Medical Imaging and Visualization Centre, Department of Radiology, Haukeland University Hospital, Jonas Liesvei 65, Postbox 7800, 5021 Bergen, Norway; 2 Section for Radiology, Department of Clinical Medicine, University of Bergen, 5020 Bergen, Norway; 3 Department of Obstetrics and Gynaecology, Odense University Hospital, Odense, Denmark; 4 Gynecological Oncology Centre, Department of Obstetrics and Gynaecology, First Faculty of Medicine, Charles University, General University Hospital in Prague, Prague, Czech Republic; 5 Department of Obstetrics and Gynaecology, Maastricht University Medical Centre, Maastricht, The Netherlands; 6 Department of Clinical Science, University of Bergen, 5020 Bergen, Norway

**Keywords:** Cervical cancer, Transvaginal ultrasound, Magnetic resonance imaging, Diffusion-weighted imaging, Positron emission tomography, Imaging biomarkers

## Abstract

**Purpose of Review:**

For uterine cervical cancer, the recently revised International Federation of Gynecology and Obstetrics (FIGO) staging system (2018) incorporates imaging and pathology assessments in its staging. In this review we summarize the reported staging performances of conventional and novel imaging methods and provide an overview of promising novel imaging methods relevant for cervical cancer patient care.

**Recent Findings:**

Diagnostic imaging during the primary diagnostic work-up is recommended to better assess tumor extent and metastatic disease and is now reflected in the 2018 FIGO stages 3C1 and 3C2 (positive pelvic and/or paraaortic lymph nodes). For pretreatment local staging, imaging by transvaginal or transrectal ultrasound (TVS, TRS) and/or magnetic resonance imaging (MRI) is instrumental to define pelvic tumor extent, including a more accurate assessment of tumor size, stromal invasion depth, and parametrial invasion. In locally advanced cervical cancer, positron emission tomography-computed tomography (PET-CT) or computed tomography (CT) is recommended, since the identification of metastatic lymph nodes and distant metastases has therapeutic consequences. Furthermore, novel imaging techniques offer visualization of microstructural and functional tumor characteristics, reportedly linked to clinical phenotype, thus with a potential for further improving risk stratification and individualization of treatment.

**Summary:**

Diagnostic imaging by MRI/TVS/TRS and PET-CT/CT is instrumental for pretreatment staging in uterine cervical cancer and guides optimal treatment strategy. Novel imaging techniques may also provide functional biomarkers with potential relevance for developing more targeted treatment strategies in cervical cancer.

## Introduction

Uterine cervical cancer is the most common gynecologic malignancy worldwide and one of the leading causes of cancer-related deaths in women, especially among women living in lower-income countries [1]. Cervical cancers, until recently, were strictly clinically staged according to the International Federation of Gynecology and Obstetrics (FIGO) system on the basis of gynecologic examination, and if needed cystoscopy, proctoscopy, colposcopy, and biopsy [2]. Since the FIGO staging revision in 2018 [3•], available imaging and pathological assessments are incorporated in stage determination. The FIGO stage is directly related to prognosis and guides the stratification of patients to different treatment regimens ranging from primary (radical) surgical resection to definitive chemoradiation or palliative chemotherapy [4].

## FIGO and TNM Staging

Until 2018, the FIGO staging system for cervical cancer was solely clinical and thus did not require information on nodal status. Furthermore, diagnostic imaging findings, known to substantiate and refine the clinical findings, were not formally incorporated in the stage. Therefore, in 2018, the European Society of Gynecological Oncology (ESGO) together with the European Society for Therapeutic Radiotherapy and Oncology (ESTRO) and the European Society for Pathology (ESP) jointly published new guidelines for the staging, treatment, and follow-up of cervical cancer patients where they recommended dual pretreatment staging by TNM and FIGO [5]. TNM staging does to a much stronger degree than the former FIGO 2009, integrate various modalities, including imaging [6], more closely reflecting the standard clinical practice in many high-income countries. With the publication of the new FIGO staging system in 2018, also FIGO acknowledges the large contribution of imaging on evaluating disease extent, with a direct consequence for treatment and prognosis. As an example, imaging singly diagnoses regional metastatic lymph nodes (FIGO stage 3C), hydronephrosis (FIGO stage 3B), and distant metastases (FIGO stage 4B), all with therapeutic consequences.

The high incidence rate of cervical cancer in lower-income countries, with more limited or at least not standard access to imaging facilities, explains why the FIGO staging system previously did not incorporate imaging findings. It has been argued that unequal staging requirements would lessen the comparability between countries and thus reduce the statistical power for global analyses. However, high-income countries have already introduced various imaging modalities at pretreatment cervical cancer staging leading to increased diagnostic accuracy and correspondingly more tailored treatment algorithms; factors that could not be weighed appropriately in the previous FIGO staging system [5]. The ESGO/ESTRO/ESP guidelines combining TNM and the clinical (2009) FIGO as well as the revised FIGO (2018) staging have aimed to bridge this gap. The FIGO (2018) staging system though does not recommend any routine (imaging) investigations, and thus, allocation to final stage in different countries will remain different. The ESGO/ESTRO/ESP guidelines, a European initiative, are stricter and state that the (TNM) staging should be based on various modalities including imaging, pathology and physical examination, documenting the modality used to determine the stage.

## Diagnostic Imaging at Primary Staging

Most centers in high-income countries routinely employ diagnostic imaging at primary work-up in cervical cancer. Accordingly, the National Cancer Comprehensive Network (NCCN) guidelines [7] for cervical cancer recommend pelvic magnetic resonance imaging (MRI), chest radiography and/or chest/abdominal/pelvic computed tomography (CT), or whole-body positron emission tomography-CT (PET-CT) in the primary diagnostic work-up to assess local tumor extent and metastatic spread. Which imaging modality each individual patient undergoes is typically guided by putative FIGO stage, perceived risk of metastatic disease, and local access to the different imaging modalities.

For assessment of local tumor extension, MRI has long been considered the imaging method of choice providing exquisite soft tissue resolution allowing an accurate assessment of tumor size, localization, and local infiltration as well as pelvic lymph node enlargement [8, 9]. Transrectal ultrasound (TRS) or transvaginal ultrasound (TVS) may also provide detailed and accurate information on local tumor extent when performed by ultrasound-trained gynecologists [10, 11].

For the assessment of pelvic and paraaortic lymph node metastases and for the detection of distant metastases in locally advanced cervical cancer, MRI and CT have been widely used [9]. However, both MRI and CT have low sensitivities for the detection of metastatic lymph nodes [9], whereas PET-CT reportedly demonstrates better sensitivity [12, 13]. This may be related to the size criterion typically employed to diagnose metastatic lymph nodes at CT and MRI, thus by definition missing the smaller metastases, and to the fact that CT and MRI are inferior to PET-CT for differentiation between metastatic enlarged nodes and hyperplastic enlarged nodes. However, PET-CT also yields low sensitivity for diagnosing small/microscopic metastases [14]. Thus, negative imaging findings for lymph node metastases based on any imaging modality do not rule out occult lymph node metastases in cervical cancer. This is clearly illustrated by a recent randomized trial reporting significant rate of upstaging (33%) after surgical staging (including paraaortic lymph node dissection) compared to clinical/radiological stage, in locally advanced cervical cancer patients (FIGO stage 2B-4) [15]. These limitations in diagnosing paraaortic lymph node metastases preoperatively are also taken into account in the ESGO-ESTRO-ESP cervical cancer guidelines (2018), which recommend surgical staging, consisting of paraaortic lymph node dissection in locally advanced cervical cancer also in patients with negative paraaortic lymph nodes on imaging [11].

It should also be noted that nonenlarged FDG-PET-CT positive lymph nodes may pose serious diagnostic challenges. These lymph nodes are normally considered for biopsy in order to confirm or rule out metastatic disease, which is necessary to enable the appropriate treatment. Unfortunately, these normal-sized lymph nodes may sometimes be hard to identify during surgery, and the biopsies may thus yield false negative results. Furthermore, whether the PET-positive lymph nodes actually have been removed at surgery may be hard to confirm, unless a new PET-CT examination is performed.

Novel functional imaging methods within ultrasound (CEUS, contrast-enhanced ultrasound), MRI (including dynamic contrast-enhanced (DCE) MRI and diffusion weighted imaging (DWI)), hybrid imaging techniques, i.e., positron emission tomography-magnetic resonance imaging (PET-MRI) as well as the use of different radiotracers for PET continue to gain further interest as promising additional imaging tools in the characterization of various cancer types, including cervical cancer [10, 12, 16–23, 24•, 25]. This interest originates in their ability to visualize and quantify functional and microstructural tumor characteristics preoperatively which have been shown to be closely associated to clinical phenotype, FIGO stage, lymph node metastases, prognostic histological tumor markers, treatment response, and outcome [10, 12, 16–23]. Thus, both conventional and functional imaging may potentially provide preoperative imaging biomarkers in cervical cancer, relevant for treatment and prognosis, and for monitoring treatment response. These biomarkers may, if further translated into the clinic, lead to substantial clinical benefit, enabling improved risk stratification and precision treatment. For example, if nodal metastasis is more accurately diagnosed preoperatively, patients can be tailored directly to their right treatment modality, avoiding surgery, and thus avoiding increased risk of long-term side effects including lymph edema through the exposure to both surgery and chemoradiation [26–28]. This will also reduce the treatment costs. Similarly, patients with tumor burden nearing the upper limit of surgical resectability will benefit from more accurate characterization and treatment planning. Finally, in patients who wish to opt for fertility sparing treatment options, the surgical feasibility and remaining functional cervical length need to be carefully assessed.

This review provides an overview of current conventional and novel imaging methods relevant for pretreatment staging and treatment planning in cervical cancer and their corresponding reported staging performances. The potential of novel functional imaging methods to yield further imaging biomarkers to individualize treatment based on improved preoperative risk stratification in cervical cancer is also discussed.

## Prognosis and Treatment in Relation to FIGO Stage

The prognosis in cervical cancer is highly dependent on FIGO stage and ranges from almost 100% 5-year disease-free survival rates for stage 1A to 5–15% for stage 4 [2, 29]. Imaging and histological findings that have recently been added in the FIGO staging system are lymph node metastases. Other factors of importance include, e.g., minimum thickness of uninvolved cervical stroma, lymphovascular space invasion (LVSI), age, comorbidities (e.g., anemia, HIV infection), and high-risk histological subtypes, e.g., adenosquamous and neuroendocrine carcinomas [29–32].

Primary treatment of cervical cancer is guided by clinical staging results and findings from diagnostic imaging [33]. For early-stage disease (FIGO (2018) stage 1A, 1B1, 1B2) treatment typically consists of surgery or definitive chemoradiation [34], the former consisting of either conization, simple hysterectomy, radical hysterectomy or fertility-sparing surgery (conization, simple trachelectomy or radical trachelectomy) typically combined with pelvic lymph node staging except in FIGO stage 1A1 with lymphovascular space invasion (LVSI)-negative tumor [34, 35]. However, as chemoradiation is equally effective in early stage disease (but rendering patients susceptible to more unpredictable long-term side effects and menopause), surgery should only be performed if no risk factors requiring adjuvant radiation treatment, including metastatic lymph nodes, are identified [26]. For fertility-sparing treatment, established eligibility requirements exist which require preoperative imaging for their assessment: histologically proven squamous cell carcinoma or usual-type (human papillomavirus-related) adenocarcinoma with size ≤ 2 cm, distance from tumor margin to internal cervical os > 1 cm and absence of suspected lymph node metastases [35]. Radical surgery may represent a therapeutic option in selected patients with tumor size > 4 cm (FIGO stage 1B3/2A2) and negative lymph nodes on radiological staging, in particular in patients without other risk factors. In locally advanced cervical cancer (FIGO stage ≥2B), definitive management with concomitant pelvic chemoradiotherapy (platinum based) and brachytherapy is the preferred treatment [5, 33, 34]. The planning of radiotherapy target volume is guided by clinical stage and very much influenced by diagnostic imaging findings that allow the delineation of local tumor extension and the identification of pelvic and paraaortic nodal involvement before radiotherapy [34, 35]. Additionally to guide target volume in locally advanced cervical cancer, paraaortic dissection may be considered before treatment for staging purposes in patients with negative paraaortic lymph nodes on imaging.

## Pelvic Tumor Extent and Distant Spread

Especially in high-income countries imaging is an essential part of the diagnostic work-up in cervical cancer and supportive to determine the TNM and revised FIGO stage with focus on tumor size, hydronephrosis, and spread beyond the pelvis. It also identifies imaging findings that yield prognostic information relevant for therapy choice, i.e., presence of lymph node metastasis and more accurate determination of tumor size. Combined with results from clinical examinations and histopathological examination of the tumor, preoperative imaging defines the optimal therapeutic strategy in cervical cancer patients.

The diagnostic performances of preoperative imaging methods for the identification of markers suggesting more aggressive disease [36, 37], including tumor size > 4 cm, deep stromal invasion (tumor invading > 2/3 of the stromal wall), parametrial infiltration, and pelvic lymph node metastases (Table 1) are critical, if these are to safely guide a tailored therapeutic approach in cervical cancer patients. Equally critical is how the reported diagnostic performance based on the different imaging methods compares with the performance of the clinical examination, serving as reference (Table 1). Importantly, treatment planning should exclusively be undertaken in gynecologic-oncological centers with comprehensive expertise in diagnosis and management of gynecologic cancers having a large volume of patients. The experience of readers, irrespective of imaging modality, is also critical for accurate pretreatment staging and assessment of treatment response.

**Table 1:** Reported diagnostic performance of pelvic imaging methods and diagnostic pelvic examinations for the assessment of large tumor size (> 4 cm), deep stromal invasion (> 2/3 of the stroma), parametrial invasion, and metastatic lymph nodes in cervical cancer

Imaging method/diagnostic examinations	Tumor size > 4 cm			Deep (> 2/3) stromal invasion			Parametrial invasion			Lymph node metastasis		
	Sens. (%)	Spec. (%)	Acc. (%)	Sens. (%)	Spec. (%)	Acc. (%)	Sens. (%)	Spec. (%)	Acc. (%)	Sens. (%)	Spec. (%)	Acc. (%)
TRUS/TVUS [10, 38–41]	78	99	95	88–91	93–97	91–93	60–83	89–100	87–99	43	96	NR
CT [13, 35, 42–44]	NR	NR	NR	NR	NR	NR	14–55	77–100	74–82	31–58	92–97	NR
Conventional MRI [10, 13, 24•, 35, 40, 42–45]	81	95	93	89	88	88	40–90	77–98	65–97	37–71	83–93	77
DWI [46, 47, 48•]	NR	NR	NR	NR	NR	NR	81–92	78–99	79–98	86	84	NR
MRI with USPIO [49]	NR	NR	NR	NR	NR	NR	NR	NR	NR	91–100	87–94	88–95
FDG PET-CT [13, 35, 43, 50]	NR	NR	NR	NR	NR	NR	NR	NR	NR	34–82	93–100	NR
FDG PET-MRI [24, 25]	NR	NR	NR	NR	NR	NR	90	94	NR	83–91	90–94	87
Sentinel node biopsy [43, 51, 52]	NR	NR	NR	NR	NR	NR	NR	NR	NR	91	100	NR
Clinical examination [41]	NR	NR	NR	NR	NR	NR	52	92	84	NR	NR	NR

*Acc.* accuracy, *CE* contrast enhanced, *CT* computed tomography, DWI diffusion weighted imaging, *FDG* fluorodeoxyglucose, *MRI* magnetic resonance imaging, *NR* not reported, *PET* positron emission tomography, *Sens.* sensitivity, *Spec.* specificity, *TRUS* transrectal ultrasound, *TVUS* transvaginal ultrasound, *USPIO* ultrasmall particles of iron oxide

### TVS/TRS

Transvaginal/transrectal ultrasound (TVS/TRS) is typically performed by the treating gynecologist with the advantage of being readily available at low cost. The transrectal approach is a preferred option over transvaginal insertion of the probe in case of bulky tumor to reduce the risk of bleeding from exophytic portion of tumor and enables better analysis of a distal part of the cervix which is often hampered by artifacts due to tumor bleeding, necrotic friable tissue, and contact between the probe and the tumor.

**Fig. 1:**
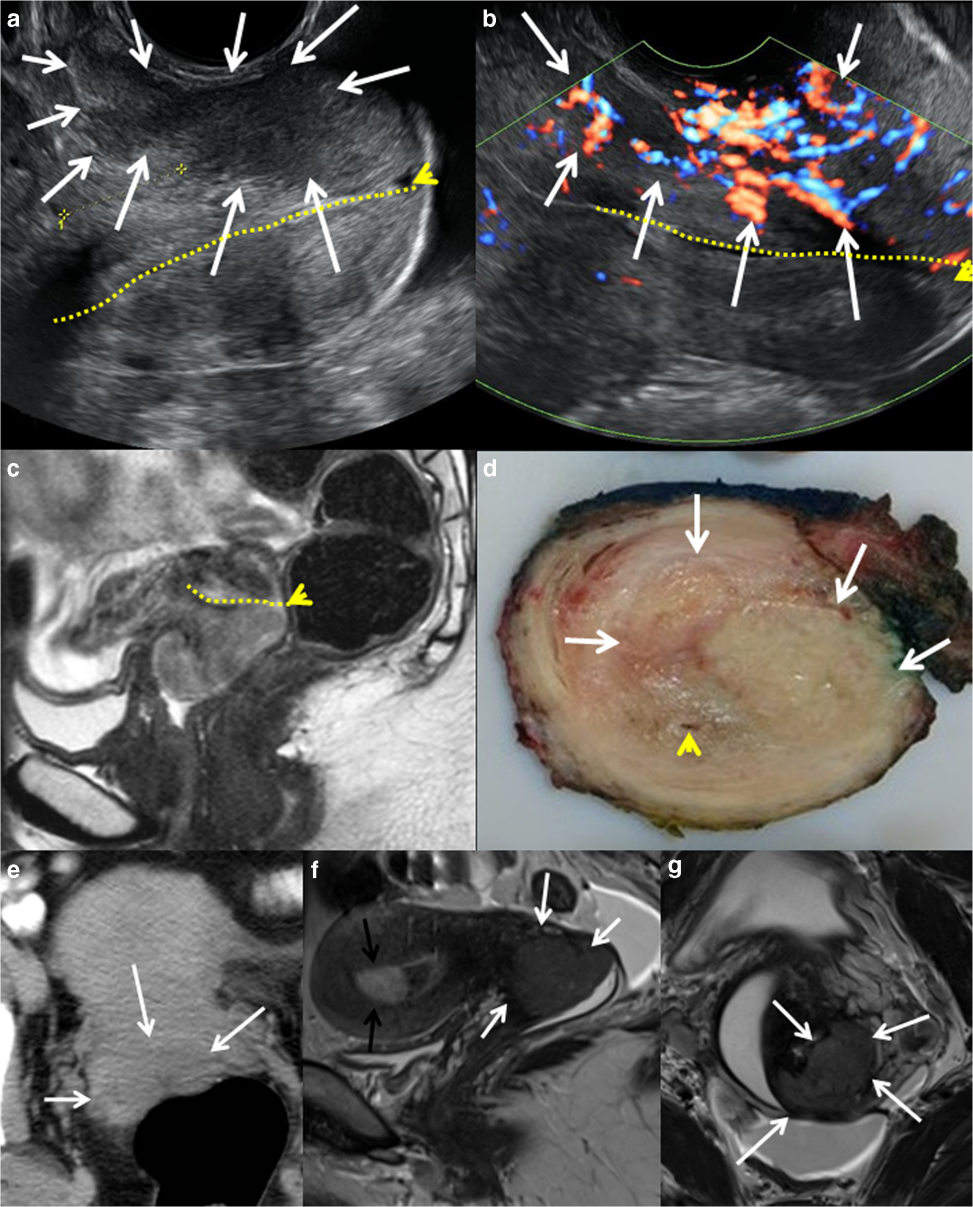
Bulky cervical cancer depicted by grayscale TRS (**a**), color Doppler TRS (**b**), and sagittal T2-weigthed MRI (**c**) in a 26-year-old patient with squamous cell carcinoma, clinical FIGO stage 1B2. The cervical cancer lesion (white arrows) appears hypoechoic on TRS (**a**) and with high perfusion on color Doppler (**b**). The same tumor is hyperintense on T2-weighted MRI (**c**). After surgical removal macroscopic transversal section (**d**) shows tumor infiltrating the anterior lip of the cervix (white arrows), while the endocervical canal (yellow line) and posterior labium is intact (yellow tip showing external cervical os). Axial contrast-enhanced CT (**e**) depicting a slightly hypodense cervical lesion (arrows) relative to the surrounding stroma in a 54-year-old patient with squamous cell carcinoma, clinical FIGO stage 1B1. Sagittal (**f**) and paraaxial (**g**) T2-weighted MRI depicting a hyperintense cervical lesion (arrows) in a 41-year-old woman with squamous cell carcinoma, clinical FIGO stage 1B1 cervical cancer (same patient as in Fig. 2 and Fig. 3a, b). The incidentally detected hyperintense lesion in the uterine cavity (**f**, black arrow) was diagnosed as a benign endometrial polyp

The cervical cancer tissue is typically depicted as hyper- or isoechoic (relative to the surrounding stroma) in adenocarcinomas and hypoechoic in squamous cell carcinomas [10, 53] (Fig. 1a). The reported diagnostic performance of TVS/TRS for the assessment of tumor size > 4 cm, deep stromal invasion (tumor invading > 2/3 of the wall), and parametrial invasion is overall quite good with reported sensitivities (specificities) [accuracies] of 78% (99%) [95%], 88–91% (93–97%) [91–93%], and 60–83% (89–100%) [87–99%], respectively [10, 38–41].

TVS/TRS, when performed at experienced centers, reportedly yields comparable diagnostic staging performance metrics to that of pelvic MRI (Table 1) [10, 35, 40, 42–44].

A European multicenter trial of early stage cervical cancer even suggests that TVS/TRS may be more accurate than MRI in detecting post-conization residual tumor and in assessing parametrial invasion [40], whereas after neoadjuvant chemotherapy a lower sensitivity of TVS/TRS than of MRI in detecting residual tumor was reported in a different single-center study [54]. Furthermore, TVS/TRS has an established role for assessing the eligibility criteria of fertility sparing treatment, with high accuracy for measuring distance from tumor to the internal cervical os and the remaining cervical length after cone biopsy. Intraoperative ultrasound may also aid the surgeon in deciding the optimal excision level securing a maximum length of tumor-free cervix for future pregnancies.

Consistent reliability of ultrasound diagnostics is, however, inherently dependent on the experience and skills of the ultrasonographer, being able to obtain representative images depicting the pathology of interest, and ultrasound is thus especially prone to interobserver variation. Its acceptance is also dependent on the technical ease of storage and retrieval of especially high-quality dynamic images, where ease of retrieval on demand is a must. Due to the small field of view and limited depth of penetration using high-frequency vaginal ultrasound probes, TVS/TRS is not considered suited for valid assessment of pelvic and paraaortic lymph node metastases (Table 1). However, with technical advances in vaginal probes allowing increasing depth of penetration, TVS/TRS may to some extent allow visualization of deeper pelvic structures, i.e., internal and external iliac lymph nodes, given that no bowel air obstructs the view.

### MRI

Pelvic magnetic resonance imaging (MRI) has long been established as a valuable imaging method in the primary diagnostic work-up of macroscopically visible cervical cancers (stage ≥1B) [55]. According to established guidelines, MRI should include at least two T2-weighed sequences in sagittal (Fig. 1c, f), axial oblique (Fig. 1g), or coronal oblique orientation in relation to the long and short axis of the uterine cervix [55]. Including an axial T1-weighted sequence from the symphysis to the left renal vein is recommended for detection of enlarged pelvic and/or abdominal lymph nodes. DCE MRI with T1-weighted series and DWI are optional sequences according to European Guidelines on cervical cancer [55]. However, these imaging methods are increasingly routinely performed at many centers and may also prove particularly valuable for patients desiring fertility preserving treatment in order to assess their eligibility [56, 57].

Cervical cancers are typically intermediate hyperintense on T2-weighted images (Fig. 1c, f–g); the sagittal plane allows evaluation of tumor extension into the uterine body or the vagina whereas the axial oblique plane is better suited to assess parametrial invasion. On noncontrast T1-weighted series, the tumor is usually isointense to the normal cervix.

**Fig. 2:**
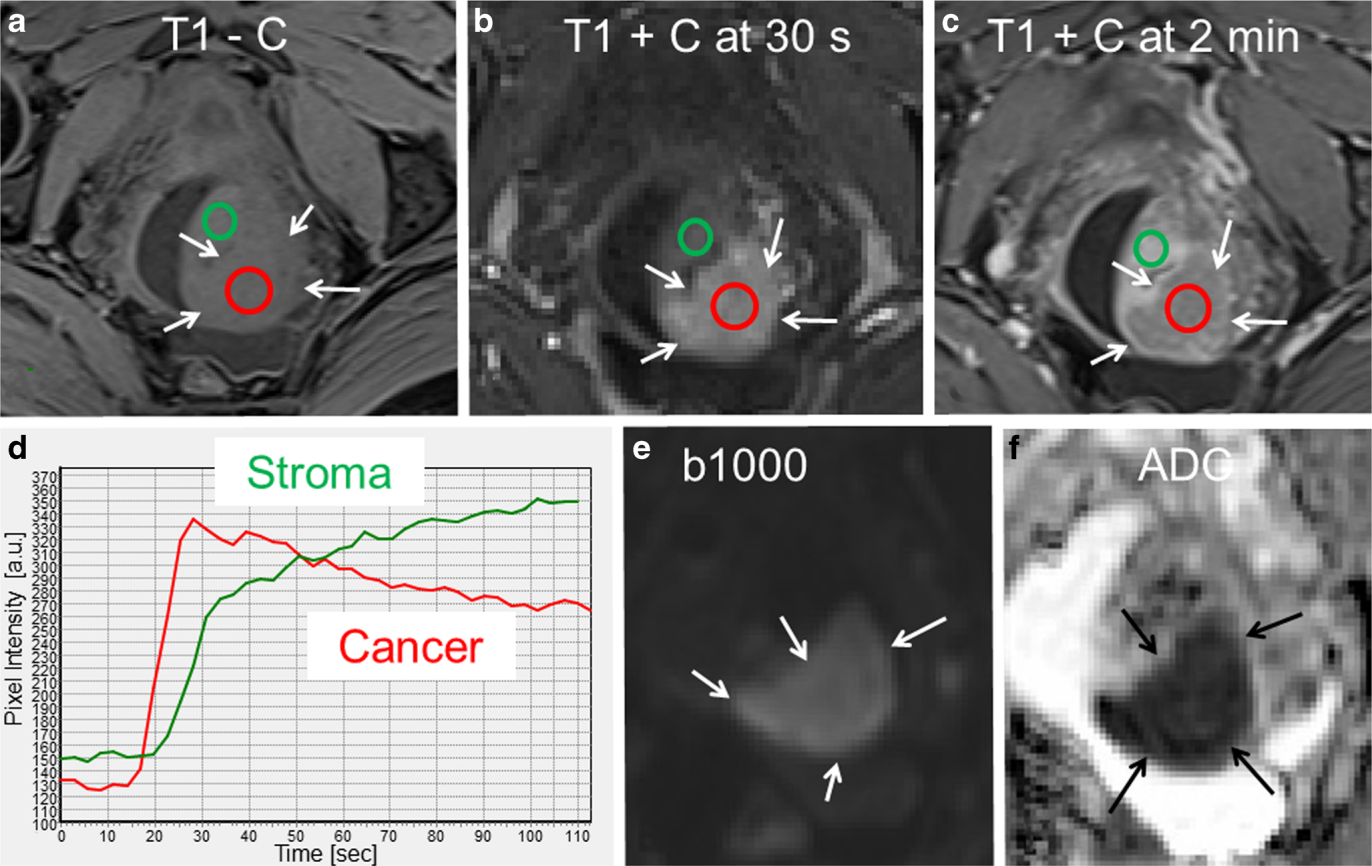
Paraaxial T1-weigthed MRI prior to contrast (**a**), at 30 s postcontrast (**b**) and 2 min postcontrast (**c**) and DWI (**e**, b = 1000 s/mm^2^) with corresponding ADC map (**f**) depicting a cervical lesion (arrows) in a 41-year-old woman diagnosed with squamous cell carcinoma, clinical FIGO stage 1B1 cervical cancer (same patient as in Figs. 1f, g and 3a, b). The cervical cancer tissue (arrows; red ROIs in **a**–**c**) is isointense relative to the normal stromal tissue (green ROIs in **a**–**c**) on T1-weighted MRI prior to contrast (**a**), hyperintense at 30 s postcontrast (**b**), and slightly hypointense at 2 min postcontrast (**c**). The corresponding time-intensity curves of cervical cancer tissue and normal stromal tissue are depicted in **d**. The tumor tissue also exhibits restricted diffusion with hyperintensity on high *b*-value DWI (**e**) and hypointensity on the corresponding ADC map (**f**). Due to MRI findings suggesting left parametrial invasion with disrupted stromal ring to the left (best seen in **c** and Fig. 1g), this patient was subjected to primary brachytherapy followed by chemotherapy (cisplatin). The patient had no signs of recurrence at 2.5 years posttreatment

On DCE-MRI, tumors typically exhibit early enhancement with hyperintensity relative to the normal cervical stroma in the arterial phase (~ 30 s postcontrast), and with early wash-out in the tumor, making it typically hypointense relative to the normal cervical stroma in the equilibrium and late equilibrium phase (~ 2 min and > 2 min post contrast) (Fig. 2a–d). Smaller tumors typically enhance homogeneously, whereas larger tumors are frequently necrotic with resulting variable enhancement, however often exhibiting an enhancing rim that facilitates tumor delineation [56]. The DCE-MRI cervical tumor enhancement pattern with characteristic early arterial enhancement in cervical cancer as opposed to delayed enhancement in endometrial cancer may also aid in differentiating between these two entities when preoperative biopsies are inconclusive [58].

DWI is an adjunctional functional MRI sequence which is increasingly employed for tumor diagnostics, also when located within the pelvis [59]. DWI depicts water mobility within tissues, a characteristic which allows it to be indirectly informative on tumor microstructure, tumor cellularity, and cellular membrane integrity. Furthermore, DWI enables quantitative assessment of diffusion properties based on the calculated apparent diffusion coefficient (ADC), which is a function of the exponential decrease in tissue signal intensity with increasing diffusion-weighting (*b*-values) [56, 60]. Cervical cancer tissue typically exhibits restricted diffusion with high signal intensity of the primary tumor and metastatic lymph nodes on high *b*-value DWI and corresponding low signal intensity on the ADC map (Fig. 2e, f).

The reported sensitivities (specificities) [accuracies] of conventional MRI (primarily based on T2-weighted sequences) for the detection of tumor size > 4 cm, deep stromal invasion, parametrial invasion, and lymph node metastases are 81% (95%) [93%], 89% (88%) [88%], 40–74% (77–98%) [70–97%], and 37–60% (92–93%) [NR], respectively (Table 1) [10, 35, 40, 42–44]. Importantly, these numbers for assessment of parametrial invasion exceed those of clinical examination with reported sensitivities (specificities) [accuracies] of 52% (92%) [84%] (Table 1) [41].

Notably, the reported diagnostic performance for the assessment of parametrial invasion and lymph node metastases has a wide range. Although DCE-MRI usually depicts the cervical tumor boundaries very precisely, it has not been shown to yield better local staging accuracy compared to that of T2-weighted images [56]. The addition of DWI, though, reportedly yields better sensitivities (specificities) [accuracies] for the detection of parametrial invasion (81% (78%) [79%]) and lymph node metastases (86% (84%) [NR]) [46, 47]. The addition of DWI has also been shown to yield higher reader confidence and better tumor delineation among the less experienced radiologist, whereas the measured maximum tumor dimensions are practically identical when derived from DWI and the conventional series [61].

The use of lymph node specific contrast agent based on ultrasmall particles of iron oxide (USPIO) has been shown to dramatically improve the diagnostic performance of MRI for the detection of metastatic lymph nodes in uterine cancer with reported sensitivity (specificity) [accuracy] of 91–100% (87–94%) [88–95%] (Table 2) [49]. Unfortunately, this contrast agent has been withdrawn by the manufacturer pending further validation before potential implementation in the clinic.

**Table 2:** Potential imaging biomarkers in cervical cancer

Imaging modality and/or parameter (i.e., imaging biomarker)	Imaging characteristics of primary tumor predicting aggressive features/disease	Possible link between imaging biomarker and tumor pathophysiology	Proposed tumor cutoffs for risk stratification
Tumor size all imaging modalities	Large tumor size predicts deep stromal invasion, parametrial involvement, lymph node metastases and poor prognosis [38, 45, 62•, 63]	Large tumor size is a marker of aggressive disease	Tumor size: > 20.5 mm predicts deep stromal invasion [38]; > 30 mm predicts parametrial invasion [45]
TVU			
Echogenicity	Isoechoic and hyperechoic tumors, relative to normal cervical stroma, are more common in adenocarcinomas while hypoechoic tumors are often found in squamous cell carcinomas [10, 53]		
Doppler parameters	Abundant vascularization is associated with aggressive disease and poor treatment response [10, 16]. Low VI predicts poor treatment response in LACC [64]	Increased vascularization is marker of aggressive disease. Tumor hypoxia predicts resistance to therapy.	PI < 0.82 predicts high-risk disease [16]
MRI			
ADC-value (based on DW MRI)	Low tumor ADC and low ADCmin predict squamous cell subtype, high grade, PMI and recurrence/metastases/poor survival [17–19, 47, 65] Textural tumor ADC features predict histologic grade and nodal metastases [66, 67]	Increased cellularity and intratumor heterogeneity of water movement predict aggressive phenotype	ADC < 0.9 for PMI [47] ADC < 0.85 and ADCmin ≤ 0.61 for poor prognosis [17, 18]
Blood flow (based on DCE-MRI)	Pretreatment tumor DCE-MRI markers reflecting reduced blood flow/contrast enhancement predict poor treatment response and survival [21, 22, 68, 69]	Tumor hypoxia is linked to therapy resistance and aggressive disease	LETV > 0.6 cm^3^ predicts poor outcome [22]; F_p_ < 50th percentile predicts poor outcome [69]
FDG PET-CT	High values of SUV_max_, SUV_mean_, MTV, and TLG in tumor and/or in lymph nodes predict advanced disease and recurrence [63, 70, 71]	Increased tumor metabolism is linked to advanced disease and aggressive phenotype	Tumor SUVmax ≥ 15.6, MTV > 48 mL, and nodal MTV > 10 mL predict poor prognosis [17, 70, 72]

*ADC* apparent diffusion coefficient (10^−3^ mm^2^/s), mean value unless otherwise specified, *ADCmin* minimum ADC value, *AP* anteroposterior, *CC* craniocaudal, *DCE* dynamic contrast enhanced, *DW* diffusion weighted, *F*_*p*_ plasma flow, *FDG* fluorodeoxyglucose, *LACC* locally advanced cervical cancer, *LETV* low-enhancing tumor volume, *LNM* lymph node metastases, *MRI* magnetic resonance imaging, *MTV* metabolic tumor volume, *NR* not reported, *PET* positron emission tomography, *PI* pulsatility index, *PMI* parametrial invasion, *SUV* standard uptake value, *TFD* tumor free distance to serosa, *TLG* total lesion glycolysis, *TV* transverse, *TVS* transvaginal ultrasound, *VI* vascularization index

### CT

In locally advanced cervical cancer or in early stage disease with suspicious lymph nodes on pelvic MRI or TVS/TRS, contrast-enhanced (CE) computed tomography (CT) of the thorax, abdomen, and pelvis is widely employed at primary diagnostic work-up for the detection of lymph node metastases and distant spread. CT images are typically acquired in the axial/transverse plane (Fig. 1e), perpendicular to the long axis of the body, and the images may be reformatted in various planes. Intravenous contrast is normally recommended, unless contraindicated.

Primary tumors, when visible on CE CT, are typically depicted as slightly hypodense or isodense relative to the surrounding stromal and myometrial tissue (Fig. 1e). For local staging, CE CT has since long been regarded inferior to MRI and TVS due to lower CT soft-tissue contrast resolution. Thus, recent indices for diagnostic performance of CT for the assessment of large tumor size and deep stromal invasion are not reported in the literature. For CT assessment of parametrial invasion and pelvic lymph node metastases, the reported sensitivities (specificities) [accuracies] are 14–55% (77–100%) [74–82%] and 31–58% (92–97%) [NR], respectively, illustrating that pelvic CT has limitations in accurately defining pelvic tumor extent in cervical cancer (Table 1) [35, 42–44].

However, as published studies including meta-analyses comparing PET-CT and CT are limited by substantial between-study heterogeneity, the new ESGO-ESTRO-ESP joint guideline concludes that there is currently insufficient evidence to justify recommending PET-CT before CT for the evaluation of paraaortic lymph node metastases in cervical cancer [11, 13, 50]. Accordingly, the new ESGO-ESTRO-ESP guideline recommends CT or PET-CT for assessment of nodal and distant disease in locally advanced cervical cancer, whereas PET-CT is preferred in patients eligible for chemoradiotherapy with curative intent [11].

### PET-CT

**Fig. 3:**
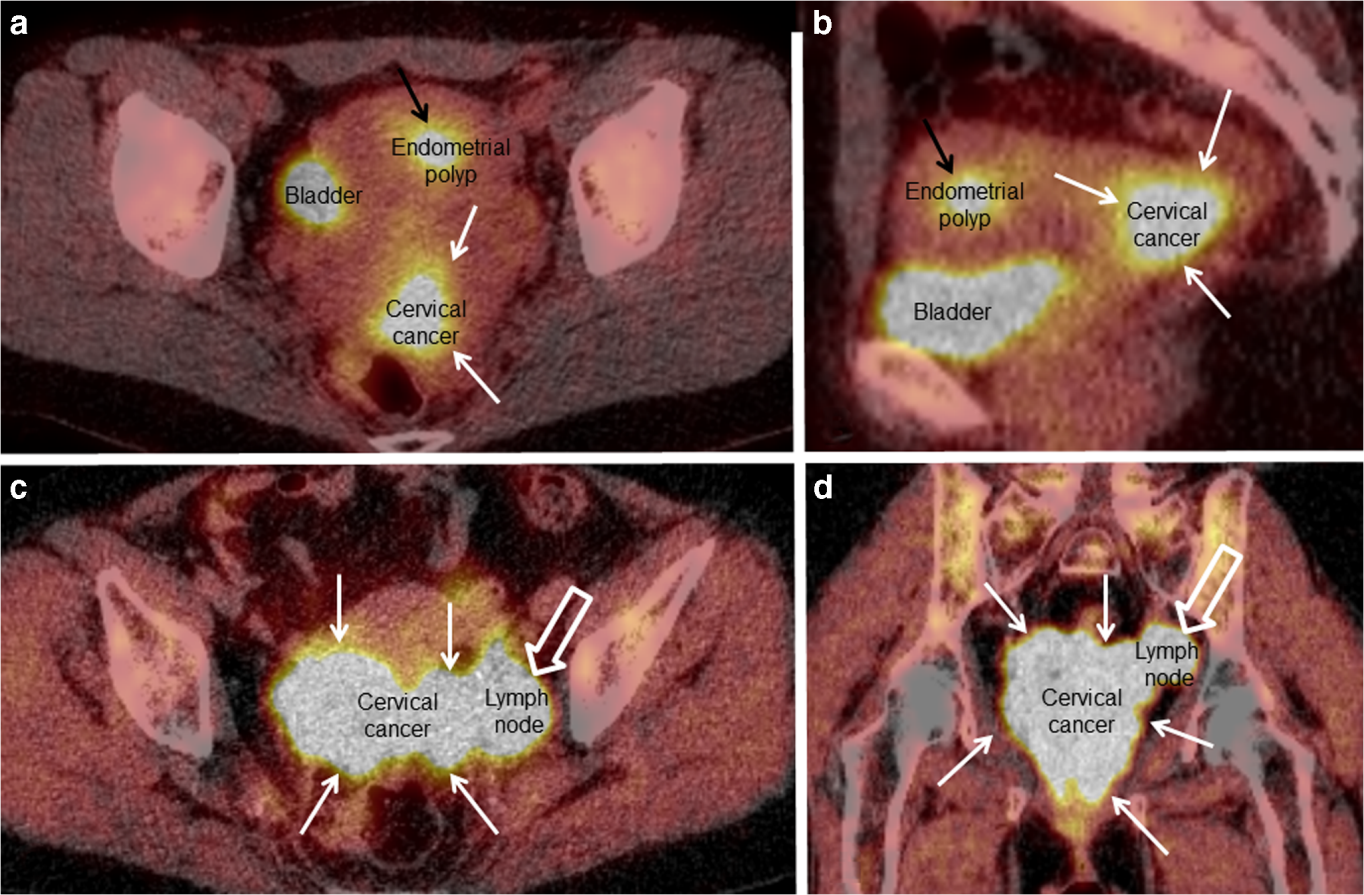
FDG PET-CT in a 41-year-old woman with squamous cell carcinoma, clinical FIGO stage 1B1 cervical cancer (**a**, **b**; same patient as in Fig. 1f, g and Fig. 2), and a 70-year-old woman with squamous cell carcinoma, clinical FIGO stage 3B cervical cancer (**c**, **d**). The primary cervical cancer lesions are typically highly FDG-avid (white arrows). The slightly FDG avid lesion (black arrow) in the uterine cavity depicted in **a** and **b** (also seen in Fig. 1f) was verified as a benign endometrial polyp. The highly FDG-avid tumor mass close to the left pelvic side wall (open white arrows; **c** and **d**) represents a metastatic iliac lymph node

Positron emission tomography-computed tomography (PET-CT) combines two imaging techniques, simultaneously visualizing both morphologic and metabolic tumor characteristics, thus allowing co-registration of structural and functional data in fused images (Fig. 3). PET-CT is increasingly employed in the preoperative staging of various cancers, including gynecologic cancers [35, 43, 50, 73, 74]. The most common radiotracer is fluorodeoxyglucose (18F-FDG), a glucose analogue that preferentially accumulates in malignant tissue due to its higher rate of glycolysis. Due to limitations of spatial resolution, FDG PET-CT is, however, unlikely to replace MRI for assessing primary local tumor extent.

For the detection of pelvic lymph node metastases FDG PET-CT, although yielding slightly lower diagnostic performance than sentinel node biopsy (Table 1) [43, 51, 52], seems to outperform conventional MRI, CT, and US, making it a very attractive noninvasive imaging method particularly in patients at high risk of metastatic disease [35, 43, 50, 52]. The reported sensitivities (specificities) of FDG PET-CT in the detection of lymph node metastases are 72–75% (93–100%) (Table 1) [35, 43, 50, 52]. The ability to correctly identify metastatic lymph nodes is, however, largely affected by lymph node size; node-based sensitivities of 100%, 67%, and 13% in metastatic nodes ≥ 10 mm, 5–9 mm, and ≤ 4 mm, respectively, have been reported in uterine cancers [14]. A down side of the method is a high percentage of nonspecific incidental findings urging additional investigations, patient distress, and raising costs.

Importantly, FDG PET-CT also provides important information about distant spread. In a multicenter trial, Gee et al. [75•] found that FDG PET-CT yielded high specificity (98%), positive predictive value (79%) and sensitivity of 55% for detecting distant metastases (defined as nonregional lymph nodes and lesions in the peritoneum, bone, liver, and lung) in patients with locoregional advanced cervical cancer. Furthermore, unexpected distant metastases were diagnosed in 14% of patients with locoregional advanced cervical cancer [75•]. Thus, the use of FDG PET-CT, particularly in high-risk cervical cancer patients, would potentially benefit patients by enabling more tailored therapy, obviating overly aggressive procedures in patients not eligible for curative therapy.

### PET-MRI

FDG PET-MRI is a novel hybrid imaging technique allowing simultaneous acquisition of MRI images, providing excellent soft-tissue contrast yielding high-resolution morphological information, combined with PET images, providing metabolic information from the same tissue [24•, 25]. In cancer patients staged by both MRI and FDG PET-CT, e.g., locally advanced cervical cancer, hybrid PET-MRI seems particularly attractive, providing multiparametric tumor imaging, with lower radiation dose exposure than that of PET-CT. A few studies exploring the value of FDG PET-MRI at primary diagnostic work-up in cervical cancer patients have been published [24•, 25]. Interestingly, one of the studies reported higher sensitivity and specificity for the identification of lymph node metastases and distant metastases based on PET-MRI compared to that of MRI alone [24•].

## Interobserver Agreement for Staging

Good interobserver agreement is crucial for the usefulness of a diagnostic test, and the interobserver agreement for any test should ideally be assessed prior to implementation in the clinic. For pretreatment staging by MRI and CT in cervical cancer, the diagnostic performance metrics as well as the interobserver agreement based on MRI are reportedly significantly better than that based on CT [44], making MRI clearly superior for cervical cancer staging. Nevertheless, some limitations in agreement are reported also for MRI with reported kappa values for staging of 0.44–0.75 [44, 76] and for the assessment of parametrial invasion of 0.70–0.75 [48•]. Interestingly, also for the assessment of clinical FIGO stage (being primarily based on pelvic examination under anesthesia), moderate (κ value of 0.49) interobserver agreement has been reported [77], illustrating that limitations in interobserver agreement are, to various extents, shared by all established methods in routine clinical use.

## Novel Imaging Methods for Staging

Current intensive research efforts on novel imaging techniques as well as new contrast agents or tracers may provide improved imaging tools, potentially enabling more accurate depiction of tumor extent and better detection of metastatic disease in cervical cancer. Furthermore, novel imaging techniques allow depiction and quantification of tumor characteristics that previously could not be assessed and that, if they prove relevant for prognosis and treatment in cervical cancer, may serve as biomarkers guiding therapy.

## Imaging Biomarkers

A biomarker is defined as a “characteristic that is objectively measured and evaluated as an indicator of a normal biologic processes, pathogenic processes, or pharmacologic response to a therapeutic intervention” [78]. Imaging-based tumor extent as well as microstructural and functional tumor characteristics may be closely linked to clinical phenotype, and may thus serve as biomarkers improving risk stratification and tailoring therapeutic strategy in cervical cancer (Table 2).

### Tumor Size

All conventional diagnostic imaging methods yield information on primary tumor size, and a large tumor has long been known to predict advanced stage and poor prognosis in cervical cancer (Table 2) [45, 62•, 63]. Two recent studies have found that primary tumor size > 20.5 mm predicts deep stromal invasion [38] and that primary tumor size > 30 mm predicts parametrial invasion [45]. Thus, by employing cutoff values for primary tumor size, pretreatment tumor size measurements may be incorporated into risk stratification models that potentially may guide therapy.

### Ultrasound Echogenicity and Doppler

Tumor echogenicity and Doppler characteristics at preoperative US may potentially provide additional information relevant for stage and prognosis in cervical cancer. Interestingly, isoechoic and hyperechoic tumors, relative to normal cervical stroma, are more common in adenocarcinomas while hypoechoic tumors are more often observed in squamous cell carcinomas [10, 53]. Abundant vascularization (Doppler ultrasound) in primary tumor is shown to be associated with high-risk histological and clinical features [16]. Furthermore, tumor US-derived 3D vascular indices prior to chemoradiation therapy are reportedly associated with treatment response in locally advanced cervical cancer [64]. Interestingly, the lower vascular indices observed in patients with poor treatment response is likely to be linked to tumor hypoxia, which is known to induce therapy resistance in various solid tumors [64].

### ADC Reflecting Microstructure

DW MRI with measurement of tumor ADC value provides additional information on tumor microstructure with potential relevance for staging and prediction of aggressive disease. In cervical cancer, low tumor ADC value and low minimum ADC value have been shown to be associated with squamous cell subtype, high grade, parametrial invasion, and poor survival [17, 19, 47, 65]. Furthermore, an early increase in tumor ADC during chemoradiation is associated with tumor size reduction and therapeutic response; thus, tumor ADC may represent a potentially useful biomarker of early treatment response [79–81].

### DCE-MRI Depicting Microvasculature

DCE MRI is a novel functional imaging technique allowing quantitative assessment of tissue perfusion and vascular permeability, enabling characterization of tumor microvasculature and the angiogenic profile of tumor tissue in vivo [82]. Tumor hypoxia, which is a characteristic feature of various solid tumors and believed to promote tumor progression and resistance to chemoradiation treatment [83, 84], may thus play a pivotal role in the pathogenic mechanisms leading to tumor growth and metastatic spread, in cervical cancer. Pretreatment DCE-MRI tumor parameters, e.g., low-enhancing tumor volume (LETV) and low-enhancing tumor fraction (LETF), have been reported to predict treatment response and survival in cervical cancer suggesting an interesting link between putative microvascular tumor hypoxia depicted by advanced imaging methods and poor radioresponsiveness in cervical cancer [22, 68]. This is supported by a recent DCE-MRI study which calculated plasma flow (F_p_), finding that low tumor F_p_ was associated with poor prognosis in cervical cancer [69].

### FDG PET-CT Depicting Metabolism

Paralleling the well-documented feasibility of FDG PET-CT for detecting regional lymph node metastases and distant spread in cervical cancer [35, 75•], the potential value of FDG PET-specific quantitative tumor parameters for predicting clinical and histologic tumor characteristics and prognosis in cervical cancer has been increasingly explored [17, 63, 70–72]. Interestingly, high values for the primary tumor metabolic PET parameters; maximum and mean standard uptake value (SUVmax and SUVmean), metabolic tumor volume (MTV), and total lesion glycolysis (TLG) have been linked to advanced stage and high-risk disease in cervical cancer [63, 70, 71, 85], suggesting that increased tumor metabolism is a marker of a biologically more aggressive cancer phenotype. Metabolic parameters may also provide markers for risk stratification that may aid in providing better individualized therapy in the future [17, 70, 72].

### In Vivo MR Spectroscopy Reflecting Biochemistry

In vivo MR spectroscopy (MRS) obtains biochemical information noninvasively from a selected volume of interest, typically tumor tissue, and signals from chemical nuclei (typically hydrogen) are registered. MRS has long been established to be a valuable adjunct to conventional MRI in the assessment of, e.g., tumors in the brain, prostate, and breast [86]. Tumor (^1^H) MRS from 3T MRI was recently reported to yield elevated tumor lipid resonance levels and characteristic spectra predicting poor prognostic HPV genotypes and persistent disease following concurrent chemoradiation in cervical cancer [87].

### Textural Imaging Features Reflecting Tumor Heterogeneity

Texture analysis is an image postprocessing technique analyzing a set of quantified metrics to assess the spatial arrangements of densities/intensities in a volume of interest. Quantitative measures of image heterogeneity have been shown to be closely linked to tissue markers of heterogeneity, hypoxia, and angiogenesis and have also been shown to predict survival for various cancers [88]. In cervical cancer, texture analysis of tumor regions of interest (ROIs) from ADC maps has been shown to yield texture markers predicting high histological grade and lymph node metastases [66]. Interestingly, high-dimensional sets of imaging features, often referred to as radiomic features, based on both MRI and PET-CT have also been shown to reveal textural markers associated with prognosis and response to therapy in cervical cancer [67, 89].

### Novel PET Tracers

A wide range of novel PET radiotracers are currently being developed with the aim of depicting and quantifying relevant biological processes and molecular targets in clinical oncology. PET imaging of cervical cancer with tracers specific for hypoxia, i.e., tracers based on fluorine-labeled nitroimidazoles (e.g., 18F-MISO and 18F-FAZA) or copper-labeled diacetyl-*bis*(N4-methylthiosemicarbazone) (60Cu-ATSM) analogues, have been tested in a few small cervical cancer patient series, hinting at a relation between increased uptake of these markers and reduced survival [90]. Nevertheless, the potential of PET-CT using hypoxia tracers in cervical cancer is still largely unexplored. Furthermore, imaging using other novel PET tracers specifically targeting cellular mechanisms (e.g., proliferation, amino acid metabolism, angiogenesis, and apoptosis), which may be dysregulated in tumor cells, may lead to better understanding of the biologic processes relevant for tumor progression and metastatic spread in cervical cancer in the future. Further testing of novel PET tracers prior to possible clinical implementation in cervical cancer patient care is thus largely awaited.

## Conclusions

Imaging during the primary diagnostic work-up in cervical cancer is essential to accurately assess tumor extent and metastatic disease, and thus select the best therapeutic option; and with the revised FIGO staging and the dual TNM/FIGO staging system according to the ESGO/ESTRO/ESP guideline its role is further emphasized. For pretreatment staging, imaging by TVS/TRS and/or MRI is instrumental to assess local pelvic tumor extent, and PET-CT or CT has a similar role to assess lymph node metastases and distant spread. Furthermore, novel imaging techniques offer visualization of microstructural and functional tumor characteristics that are linked to clinical phenotype, thus with a potential for improving risk stratification and treatment. Novel imaging biomarkers should, however, be thoroughly assessed for reproducibility and studied in combination with currently standardly applied biomarkers in cervical cancer; only then the potential added value of new imaging biomarkers for cervical cancer patient care in the future can be fully assessed.
